# The impact of toxic trolling comments on anti-vaccine YouTube videos

**DOI:** 10.1038/s41598-024-54925-w

**Published:** 2024-03-01

**Authors:** Kunihiro Miyazaki, Takayuki Uchiba, Haewoon Kwak, Jisun An, Kazutoshi Sasahara

**Affiliations:** 1 Luddy School of Informatics, Computing, and Engineering, Indiana University Bloomington, Bloomington, IN USA; 2 Sugakubunka Co., Ltd., Tokyo, Japan; 3 School of Environment and Society, Tokyo Institute of Technology, Tokyo, Japan

**Keywords:** Toxic comment, Fearful comment, YouTube videos, Vaccine hesitancy, Emotional contagion, Information technology, Human behaviour

## Abstract

Anti-vaccine trolling on video-hosting websites hinders efforts to increase vaccination rates by using toxic language and threatening claims to intimidate people and promote vaccine hesitancy. However, there is a shortage of research investigating the effects of toxic messages on these platforms. This study focused on YouTube anti-vaccine videos and examined the relationship between toxicity and fear in the comment section of these videos. We discovered that highly liked toxic comments were associated with a significant level of fear in subsequent comments. Moreover, we found complex patterns of contagion between toxicity and fear in the comments. These findings suggest that initial troll comments can evoke negative emotions in viewers, potentially fueling vaccine hesitancy. Our research bears essential implications for managing public health messaging and online communities, particularly in moderating fear-mongering messages about vaccines on social media.

## Introduction

In the face of pandemics, it is crucial to mitigate unnecessary vaccine hesitancy. The efficacy of vaccines has been established through numerous doses administrated, resulting in the preservation of countless lives^1^. However, despite these successes, anti-vaccine sentiment persists and continues to gain momentum^2^. The anti-vaccine movement may lead to widespread vaccine hesitancy, resulting in decreased vaccination rates and ultimately reducing the overall societal benefit^3^.

Social media platforms have recently become the main battleground of the anti-vaccine movement to disseminate their belief^3,4^. In these platforms, anti-vaccine groups often use toxic messaging as part of their communication strategy^5^, and it is postulated that these toxic claims contribute to the exacerbation of anti-vaccine hesitancy. Psychological experiments have established that emotionally charged and negative messages within an anti-vaccine context can incline people toward vaccine aversion^6^. Recent studies found that anti-vaccine groups exhibit a higher degree of toxicity on social media compared to other groups, such as neutral and pro-vaccine groups^7^. However, there is currently a lack of empirical knowledge on the spillover effects of such toxic messages on social media.

This study focuses on assessing the impact of toxic comments on viewers of anti-vaccine YouTube videos. Specifically, we aim to determine whether initial toxic comments are associated with the degree of fear expressed in subsequent comments. YouTube is one of the most widely visited websites in the world^8^ and has become a primary source of information for many individuals regarding pandemics and vaccines^9,10^. The comment section on YouTube videos not only provides feedback for video creators^11^, but also serves as a venue for communication and information sharing among viewers^12,13^, providing a new experience for video audiences that is different from traditional media such as television programming^14^. Moreover, comments on online content are known to shape viewers’ perceptions of the content itself^15,16^. However, the comment section is often plagued by uncivil comments^17–19^, particularly on anti-vaccine videos^20^.

Fear plays a crucial role in understanding vaccine hesitancy, especially as it relates to the impact of anti-vaccine videos^21^. Fear is also known to be an emotion associated with immediate reactions^22^, which are often analyzed in social media analysis in recent years^23^, making it an appropriate emotion for analyzing comments based on viewing videos and other comments. Empirical and quantitative evidence of the association of toxic comments with other users’ emotion would provide valuable insights for social media platforms to effectively moderate content and maintain a safe and informative environment.

The concept of ‘emotional contagion’ highlights such transfer of emotions from one person to another, describing how it impacts their emotional state. This phenomenon, well-documented in both face-to-face^24^ and online interactions^25–27^, indicates that exposure to certain emotional tones in messages can trigger a cascade of posts with similar emotions. Emotional contagion is understood through social and psychological traits like imitation and mimicry, explaining how individuals absorb others’ cognitive experiences in social interactions^28^. Particularly relevant is the observation that online toxicity can incite fear, a connection especially pertinent in the context of vaccine hesitancy. While the mechanisms of emotional contagion in spreading fear are understood in a general sense, the specific interplay between online toxicity and the propagation of fear related to vaccine hesitancy remains less explored. This gap in understanding is significant, as elucidating this link could enhance strategies for risk communication and online content moderation, particularly in managing the emotional impact of toxic messages on public health perceptions.

In this work, we conducted a quantitative analysis to examine the relationship between toxicity and fear in the comment sections of YouTube videos related to anti-vaccine content. Our dataset comprised 484 anti-vaccine videos and 414,436 corresponding comments, which were collected in previous studies^29^. Our analysis revealed that fear and toxicity tend to co-occur in the comment sections at the video level, even after controlling for other video-specific factors such as topics and emotional tones of the title, description, and transcripts. Moreover, we observed an association between the toxicity of early comments and the fear expressed in subsequent comments. In particular, the toxicity of early comments that received a high number of likes was significantly associated with the fear expressed in later comments.

## Results

### Prevalence of fear and toxicity in YouTube comments

**Figure 1:**
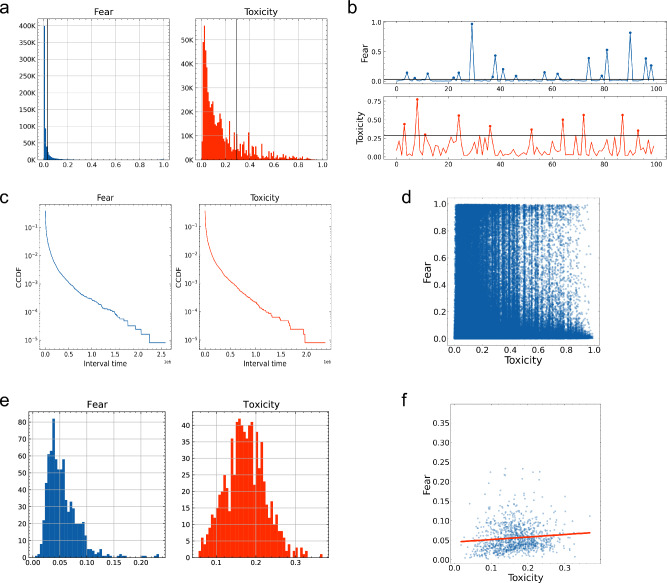
Fear and toxicity in YouTube comments at the comment and video levels. (**a**) Distribution of fear and toxicity scores for each comment, with the top 20th percentile thresholds indicated by vertical lines. Both distributions exhibit strong skewness, suggesting that many comments possess low values. (**b**) Time series of fear and toxicity scores for the first 100 comments on a specific video. The horizontal lines correspond to the 20th percentile thresholds from (**a**), and the data points surpassing these thresholds are marked. (**c**) A log-log plot of CCDFs indicating the probability of the interval time between all highly fearful and highly toxic comments at the comment level, which indicates that this interval time adheres to a heavy-tailed distribution. (**d**) Scatterplot of fear and toxicity scores per comment. (**e**) Distribution of average fear and toxicity scores per video. (**f**) Scatter plots of average fear and toxicity scores per video. The red line shows a regression line. The Pearson correlation coefficient between toxicity and fear was 0.10 ($p=0.00$), the coefficient of the single regression analysis was 0.06, and the distance correlation was 0.16.

Our objective was to assess the effects of trolling comments on anti-vaccine videos, specifically examining whether toxicity in comments for a video is associated with the level of fear expressed in the comments for the same video on YouTube. We first analysed the relationship between toxicity and fear at the video level. We employed a machine learning approach—Google’s Perspective API and a RoBERTa-based model—to quantify fear and toxicity levels in each comment, and we computed their mean for each video (see “Methods”). Our results demonstrate that highly fearful and highly toxic comments only constitute a portion of all comments. As depicted in Fig. 1a, the top 20th percentile comments have fear and toxicity scores of 0.03 and 0.29, respectively, on a 0–1 scale. These scores illustrate highly skewed distributions, with a narrow dynamic range for fear and a wide dynamic range for toxicity.

We then investigated the temporal dynamics of fear and toxicity within comment sections. Figure 1b provides an example of the absence of evident temporal patterns for fear and toxicity, where highly toxic and highly fearful comments appeared sporadically and abruptly. We also analysed the temporal intermittency of such highly toxic and highly fearful comments and found a heavy-tailed distribution for the intervals between highly toxic and highly fearful comments (Fig. 1c)^30^.

One may question whether fear and toxicity are inherently correlated within an individual comment. By analysing the scatter plot of fear and toxicity scores for each comment (Fig. 1d), we found that this is not the case. To summarise the findings thus far, both toxicity and fear in comments exhibit burst-like dynamics; however, they do not have a one-to-one correspondence and are not the same signal.

### Factors that elicit fear in YouTube comments

We quantified the distributions of fear and toxicity and their correlation in comments at the video level. Figure 1e shows the distribution of average fear and toxicity scores for comments on each video. These distributions differ from those at the comment level (Fig. 1a) and more closely resemble a Gaussian distribution. Figure 1f shows a scatter plot of fear and toxicity scores, indicating a weak correlation between average fear and toxicity scores at the video level. Based on these findings, we decided to focus on the average fear and toxicity scores compiled across entire or partial comment sections as significant metrics^31^.

Next, we examined the features associated with fear at the video level. To account for potential covariates, we conducted an ordinary least square (OLS) regression analysis with the videos as data points and the average fear of comments per video as the dependent variable. The independent variables in the regression analysis include the video’s base features (e.g., view counts), the video’s emotion-related features (e.g., fear score in a title), the video’s topics, and the comment features (e.g., fear, toxicity), all detailed in “Methods”.

**Figure 2:**
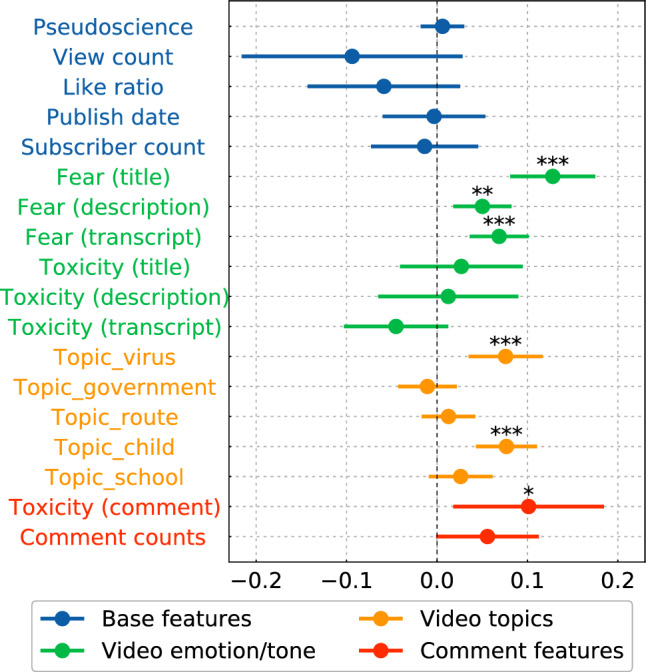
Results for video-level regression. (**a**) The coefficient of variables with 95% CIs. The stars indicate the *p* values of the t-test: *** for $p < 0.001$, ** for $p < 0.01$, and * for $p < 0.05$. The model intercept parameter is not shown.

Figure 2 shows the results of the regression analysis at the video level. Notably, the analysis identified that average toxicity is a significant variable even when controlling for other variables, implying a strong association between toxicity and fear in comments aggregated at the video level. Since the fear and toxicity scores are all on a scale from 0 to 1, the coefficients of the regression indicate how much fear increases when the toxicity score increases from 0 to 1. A large and significant cofficient indicates that the variable is correlated with the degree of toxicity in the comment section of the video. Additionally, we found that fear in comments was significantly associated with the topics of viruses and children’s diseases, which aligns with previous research linking these topics to fear among anti-vaccine groups^5,32^. Furthermore, fear in the title, description, and transcript is positively associated with fear in comments, which supports previous research indicating that the emotional content of videos can be associated with viewers’ emotions^33^. By contrast, the analysis revealed the toxicity of video content is only minimally related to fear in comments. The analysis did not reveal any significant relationship between pseudoscience and fear in comments, suggesting that the scientific nature of the content does not significantly affect fear in comments. See Supplementary Information for models with some features ablated.

### Association of early toxic comments with fear towards subsequent interactions

**Figure 3:**
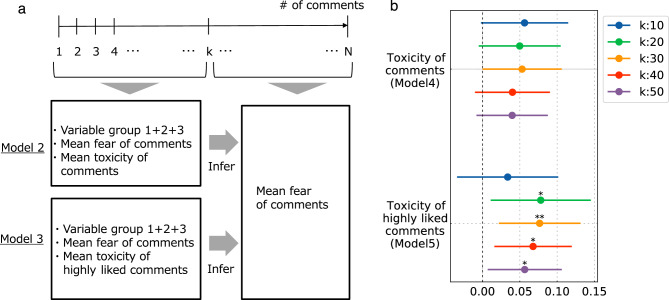
Measuring the association of toxicity of early comments with the fear in later comments. (**a**) Illustration of the problem setting. *N* comments in chronological order for a given video are divided into early and later halves, separated by *k*. Then, the average fear of comments in the comment range is predicted by the variables noted in Model 4 and Model 5, respectively, and the coefficients are obtained. (**b**) Forest plots showing the coefficients of average toxicity of comments and highly liked comments across window size $k=\{10,20,30,40,50\}$. Both are positive regardless of *k*, but only the mean toxicity of highly liked comments is largely significant. The average toxicity of highly liked comments has a high coefficient compared to the average toxicity of all comments (1.3 times higher in the average value in the five windows).

Lastly, we explored the impact of early toxic comments on subsequent comments, focusing on emotional aspects. Based on previous research that demonstrated a connection between early comment features and later sentiments in comments^34^, we employed a similar approach. A key factor to consider is the window size of comments, which sets the threshold for determining the number of early comments (Fig. 3). We compared various window sizes, with $k=\{10,20,30,40,50\}$ to gauge their effects. The objective was to calculate the average toxicity and fear in comments within these window sizes (see “Methods”), and subsequently incorporate the variable groups 1, 2, and 3 features used in the previous regression analysis to estimate the average fear in comments following the threshold *k* (Model 4 in Fig. 3a).

Considering that YouTube comments are not necessarily arranged in chronological order, we included not only the recency of comments but also their engagement, specifically the number of ‘likes’, which highly affects the order of comments. The number of likes is crucial when assessing the impact of toxic comments because the higher the value, the more likely a comment is to appear at the top of the comment list and, consequently, has a greater likelihood of influencing other comments^19,35^. In this study, we aimed to account for the effect of highly liked comments. Therefore, we used the average toxicity of comments within window *k* that have a like count in the top 20th percentile or higher, as the explanatory variable (i.e., the toxicity of *highly liked* comments, see “Methods”) (Model 5 in Fig. 3a). This approach replaced the use of the average toxicity of comments within window *k* in Model 4.

Figure 3b shows the coefficients for all comments (top) and highly liked comments (bottom) across the window sizes $k=\{10,20,30,40,50\}$ in the regression analysis. For all comments and after controlling for fear in early comments, the toxicity of early comments is slightly positively related to fear in later comments across all window sizes (*k*) (but not significant), after controlling for fear in early comments. This result suggests that early toxicity is associated with later fear independently of early fear. Note that early fear is strongly associated with later fear, confirming the contagion of homogeneous emotions. Looking at the toxicity of highly liked comments at the bottom of Fig. 3b, we can also see that only the coefficients for the toxicity of highly liked comments are significant (4 out of 5 cases). Moreover, the toxicity of highly liked comments has a particularly large coefficient (about 1.3 times), indicating that the association of the liked comments is stronger than all comments. It should also be noted that in the regression analysis, the other video-related variables were controlled, as described in “Factors that elicit fear in YouTube comments”, suggesting that emotional contagion was likely to occur between comments.

**Figure 4:**
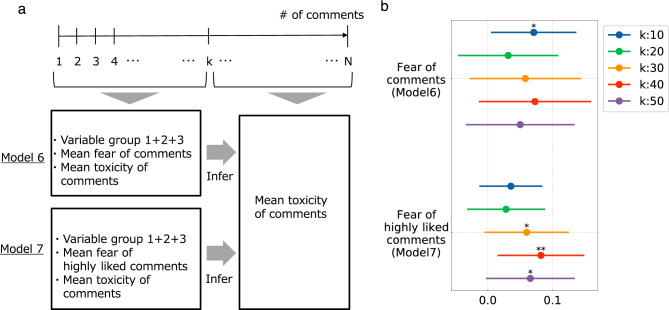
Measuring the association of the fear of early comments with the toxicity in later comments. (**a**) Illustration of the problem set. *N* comments in chronological order for a given video are divided into early and later halves, separated by *k*. Then, the mean fear in comments in the comment range is inferred by the variables noted in Model 6 and Model 7, respectively, and the coefficients are obtained. (**b**) Forest plots showing the coefficients of the fear in comments and the fear in highly liked comments, for $k=\{10,20,30,40,50\}$. Only the coefficients for fear in highly liked comments are largely significant (3 out of 5 cases).

One might wonder whether there is the opposite direction of the effect, i.e., is early fear associated with later toxicity in comments? To answer this question, we examined two more models—model 6 and model 7. In both models, we assigned the ‘mean toxicity of later comments’ as a dependent variable, and in model 7, we modified model 5 by replacing the ‘average toxicity of highly liked early comments’ with the ‘mean fear of highly liked early comments’ in the independent variable (Fig. 4a). Figure 4b suggests that the coefficients for fear in early comments are all positive. Also, the coefficients for fear of highly liked comments are largely significant (4 out of 5 cases). These findings indicate that in the comment section of anti-vaccine YouTube videos, the influence of the toxicity and fear in comments is bidirectional, with the toxicity of early highly liked comments having an influence on the fear in subsequent comments and vice versa.

## Discussion

The dissemination of fear-inducing messages about vaccines contributes to decreased vaccination rates and is a major concern. Despite the widespread presence of toxic comments from anti-vaccinationists on social media, there is a lack of empirical evidence demonstrating the association of these comments with inducing fear in other platform users. To bridge the gap, we analysed YouTube comments on anti-vaccine videos to investigate the relationship between toxicity and fear. We first identified the lack of relationship between toxicity and fear in individual comments. This is because if toxicity and fear in individual comments are strongly correlated or equivalent, the subsequent results at the aggregated level would be trivial. We then focused on both the video and the comment levels, specifically, early and later comments, while controlling for other relevant variables, to gain insights into the association between toxicity and fear in comments.

Our findings demonstrate a substantial connection between toxicity and fear in YouTube comments when analysed at the video level. Despite a lack of relationship between toxicity and fear in individual comments, aggregating them at the video level uncovers a notable association, even after controlling for other variables. This suggests that toxicity and fear cooccur within the comment sections. The phenomenon of emotional contagion, in which fear in a video’s title, description, and transcripts correlates with fear in comments, highlights the association of emotions in video content. The results regarding video topics were consistent with previous studies, which showed significant relationships between fear in comments and subjects such as viruses and children’s diseases^5,32^. However, our analysis found little effect of the video’s pseudoscience content on fear in comments. Furthermore, we observed a slight but significant association between the toxicity in early comments and fear in later comments, notably stronger (1.3 times on average and significant in 4 out of 5 cases) for highly liked early comments. These findings confirm the contagion of heterogeneous characteristics (i.e., toxicity and fear) in YouTube comment sections, suggesting the need for more aggressive moderation of highly toxic comments.

The results of this study have important implications for moderation policies on online platforms. Conventionally, the focus on toxicity in online comments has centered on its direct impact on the target of the message, such as the owner of the video. However, this study uncovers a third-person effect^36^, highlighting how comments also siginificantly associate with the emotions of other viewers. As commenting on various online content, such as videos, news, and e-commerce, profoundly impacts user experiences, it is imperative for platform providers to consider the wider effects of toxic messages. A key finding was the greater association of toxicity in *highly liked* comments, which were approximately 30% more influential than in ordinary comments. Many online platforms employ systems for rating user comments, and those with more likes often receive increased visibility. However, if these highly liked comments contain high levels of toxicity, their negative correlation could be substantial. This calls for a re-evaluation of the policies and algorithms that amplify the visibility of liked comments on online platforms. For example, platforms could remove highly toxic comments, or at minimum, place them lower in the display order, regardless of their like count.

It is important to acknowledge the limitations of this study. First, our measurement of fear in comments on anti-vaccine videos was used as a proxy measure and did not necessarily indicate fear or hesitation towards vaccines themselves. Further research could employ an aspect-based sentiment analysis^37^ and stance detection approach^38^ to extract more specific fear towards vaccines. However, these methods might be less accurate if direct references to vaccines are not present in the target text. Also, although fear is an important emotion regarding vaccine hesitancy^21^, other emotions can also be involved, such as anxiety and disgust. Therefore, it is important to acknowledge the limitations of this study. We performed an additional analysis in our video-level regression analysis, replacing the emotion of fear, a dependent variable, with the other six emotions obtainable in the RoBERTa model. As a result, the coefficients of toxicity in the comments were significantly associated with “anger” and “disgust” and higher than the coefficients of “fear”. On the other hand, the other emotions “joy”, “neutral”, and “surprise” were significantly negatively correlated, while “sadness” was not significant. As expected, negative emotions in the comment section were correlated with toxicity in the comment section (see Supplementary Information). In addition, anxiety is said to be associated with more long-term concerns, while fear is an emotion associated with more immediate reactions^22^, which might affect people’s stance on vaccines in a different way, but we keep this topic for future research. Second, our study does not establish a strict causal relationship between toxicity and fear in the comment section. While our goal was to analyse the association of toxicity with fear in comments, it would be neither ethical nor feasible to conduct field experiments. We considered alternative approaches, such as a regression discontinuity design, but their implementation was difficult due to the absence of clear trends in our time series data. Third, in the regression analysis, we used as much meta and textual information as we could think of for videos, but not image or audio information. These information may affect viewers’ emotions, and future research can incorporate them in their analysis. Consequently, we divided the time series into two parts using thresholds and compared their properties. Additionally, we cannot entirely dismiss the possibility of algorithmic bias. The videos in our sample were selected based on YouTube’s search and recommendation algorithms, which can influence the results, although we took steps to avoid “personalised” recommendations^29^. Lastly, the order in which comments are displayed on YouTube is influenced by multiple factors, such as the timing of viewing and the presence or absence of comments. We did not account for these factors in this study, because we could not fully reverse-engineer YouTube’s comment-sorting algorithm. Nevertheless, existing research indicates that the influence of ‘likes’ on comments is a primary factor influencing their display order^19,35^. Given this, by incorporating the ‘likes’ factor into our model, we offer an analysis aligning with the real-world scenario despite the simplifications.

## Methods

### Data collection and treatment

The data for this study were sourced from previously conducted research on anti-vaccine videos^29^. The data were collected from YouTube and comprised of videos labeled as ‘science’, ‘pseudoscience’, and ‘irrelevant’ using crowdsourcing. The annotations were completed by three annotators assigned to each video through the Appen crowdsourcing service. After consolidating them into two classes: “pseudoscience” and “others”, the annotations from crowdsourcing achieved a high level of agreement (F1 score: 0.92) with the annotations of the original paper’s authors. In this study, we also adopted this two-class classification because we considered the “pseudoscience” type of videos to be influential in shaping comments that expressed fear. The data collection process involved the initial identification of 346 videos using keywords “anti-vaccination” and “anti-vaxx”, followed by the collection of 1,759 recommended videos accompanying the initial videos. The resulting dataset, labeled as “anti-vaccine”, is publicly available, including annotations^29^. For the analysis, we used videos with at least 100 comments and available transcripts, which resulted in 484 videos and 414,436 comments. These transcripts can be generated either manually or automatically on YouTube^39^.

### Measuring toxicity in comments

The degree of toxicity in comments was measured using Google’s Perspective API^40^, a widely utilised tool in online abuse and harassment research. The definition of a toxic comment is “a rude, disrespectful, or unreasonable comment that is likely to make you leave a discussion”^40^. Such comments include offensive to others, negative, or hateful. The predictive model is trained using comments from forums such as Wikipedia and The New York Times, along with human annotation. This score is suitable for our analysis, which focuses on offensive comments related to anti-vaccination discussions. Specifically, this API has been applied to texts from various social media platforms, such as YouTube^19^, Twitter^41,42^, and Reddit^43^ providing a toxicity score on a scale from 0 to 1.

### Measuring fear in comments

The quantification of fear in each comment was assessed utilising a RoBERTa-based model^44^. This model, distilled from RoBERTa and fine-tuned with six datasets, was designed to predict Ekman’s six basic emotions, including fear, and a neutral class (seven classes in total). The model can assign probabilities of each emotion to each text, ranging from 0 to 1. We considered this probability as the emotion score for the text.

To validate the accuracy of the model, we compared its results with those of the Linguistic Inquiry and Word Count (LIWC)^45^. The LIWC, a tool for analysing emotions in text based on psychological categories, assigns emotions to registered words. We analysed the correlation of fear with anxiety and negative emotion, which encompasses anxiety, anger, and sadness. This verification approach, although automated, bears a resemblance to manual methods. The results showed a correlation coefficient of 0.473 ($p=0.000$) for the emotion of anxiety, and 0.228 (p = 0.000) for the negative emotion, indicating a significant correlation for both. Note that we did not use the LIWC for our main analysis due to our aim to quantify all comments. The LIWC dictionary measures sentiment in sentences that contain the registered words, and often resulting in null sentiment for many texts. In contrast, deep learning-based approaches, such as the one employed in this study, aim to capture subtle nuances in sentences and make predictions of the emotions expressed in the text. Although the decision criteria used by these models are less transparent compared to dictionary-based methods such as the LIWC, we considered them to be more appropriate for our objective of assessing the relationship between emotions at the level of individual comments. Also, the absence of a sentiment explicitly targeting *fear* in the LIWC influenced our decision to employ a deep learning-based approach.

### Topic identification

For the extraction of topics from transcript texts, we employed a biterm topic model (BTM)^46^, a methodology acknowledged to be more effective for short text than other widely-used topic models, such as Latent Dirichlet Allocation (LDA). The input for the BTM was limited to nouns^47^ after filtering with stopwords. Through the comparison of perplexity scores, the number of topics was determined to be six^48^. Once the topic model was constructed, each comment was assigned to one of the six topics as a dummy variable. We determined that assigning one topic with the highest probability of belonging to one comment was sufficient after manual inspection. The labels of the six topics were virus, government, others, route, child, and school. We set the ‘others’ topic as a base topic for the regression analysis. A summary of the six topics, their associated keywords, and the distribution of comments among each topic is provided in Supplementary Information.

### Like ratio

In the regression analysis at the video level, the like ratio was calculated for each video. This calculation was based on the number of likes for that video divided by the total number of likes and dislikes:

1 $$\begin{aligned} Like\;ratio = \frac{Num_{like}}{Num_{like} + Num_{dislike}}. \end{aligned}$$

### Regression at the video level

We grouped the independent variables in the regression model into four categories:

- Variable group 1: Base features of the videos
  - *View counts* (logged numerical value) This is considered a proxy indicator for the catchiness and interestingness of the video.
  - *Like ratio* (numerical value) This is an indicator of how well a video is supported by viewers (see “Methods” for calculation).
  - *Pseudoscience* (binary) This label is annotated in^29^ and indicates whether the content of the video is based on pseudoscience (see “Methods” about data collection).
  - *Publish date* (numerical value) This is how late the video is published (see “Methods” for calculation).
  - *Subscriber count* (logged numerical value) This is an indicator of how popular the video creator is.
- Variable group 2: Video emotion/tone
  - *Fear (title, description, transcript)* (numerical value) These are the fear scores of the text information of the titles, descriptions, and transcripts, respectively, for each video quantified by a machine learning model^44^ (see “Methods”).
  - *Toxicity (title, description, transcript)* (numerical value) These are the toxicity scores of the text information of the titles, descriptions, and transcripts, respectively, for each video quantified by a machine learning model^40^ (see “Methods”).
- Variable group 3: Video topics
  - *Topics (transcript)* (one-hot dummy encoding) This is a categorisation of videos using a topic model based on the text information of the transcripts (see “Methods” for details). The topics are divided into six groups: virus itself (*Topic_virus*), government (*Topic_government*), route of infection (*Topic_route*), child disease (*Topic_child*), school (*Topic_school*), and unrecognisable (*Topic_others*). We set *Topic_others* as the base topic and developed binary variables for the remaining five topics (see Supplementary Information* for stats and representative words for each topic).
- Variable group 4: Comment features
  - *Toxicity (comment)* (numerical value) This is the mean value of the toxicity of comments for each video.
  - *Comment counts* (logged numerical value) This is the number of comments for each video.

To avoid multicollinearity, we considered variables with a Variance Inflation Factor (VIF) less than four. Min-max scaling from 0 to 1 was conducted for all variables to compare the coefficients of the independent variables.

### Ethical approval

 In this analysis, we adhere to ethical research practices and prioritise data privacy. No personal information was included in the manuscript, specifically avoiding the inclusion of any information that could identify individuals responsible for making toxic comments.

### Supplementary Information

Supplementary Information.

## Data Availability

We used the open dataset that can be found here https://zenodo.org/record/4769731.

## Code availability

The code and supplemental dataset are available here https://github.com/Mmichio/youtube_troll_comments_public.
