# Publisher Correction: The impact of toxic trolling comments on anti-vaccine YouTube videos

**DOI:** 10.1038/s41598-024-57783-8

**Published:** 2024-03-26

**Authors:** Kunihiro Miyazaki, Takayuki Uchiba, Haewoon Kwak, Jisun An, Kazutoshi Sasahara

**Affiliations:** 1 Luddy School of Informatics, Computing, and Engineering, Indiana University Bloomington, Bloomington, IN USA; 2 Sugakubunka Co., Ltd., Tokyo, Japan; 3 School of Environment and Society, Tokyo Institute of Technology, Tokyo, Japan

Correction to: *Scientific Reports* 10.1038/s41598-024-54925-w, published online 01 March 2024

In the original version of the Article, Figure 4 was a duplication of Figure 3. The original Figures 3 and 4 and accompanying legends appear below.

The original Article has been corrected.

**Figure 3:**
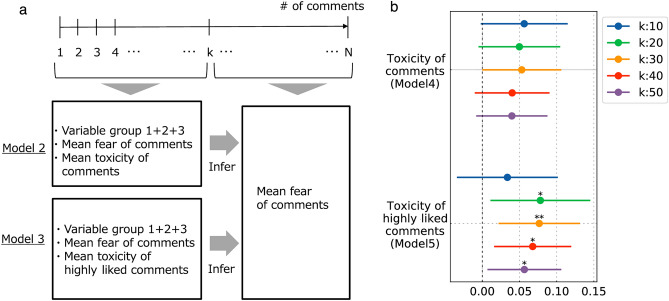
Measuring the association of toxicity of early comments with the fear in later comments. (**a**) Illustration of the problem setting. *N* comments in chronological order for a given video are divided into early and later halves, separated by *k*. Then, the average fear of comments in the comment range is predicted by the variables noted in Model 4 and Model 5, respectively, and the coefficients are obtained. (**b**) Forest plots showing the coefficients of average toxicity of comments and highly liked comments across window size $k=\{\mathrm{10,20,30,40,50}\}$. Both are positive regardless of *k*, but only the mean toxicity of highly liked comments is largely significant. The average toxicity of highly liked comments has a high coefficient compared to the average toxicity of all comments (1.3 times higher in the average value in the five windows).

**Figure 4:**
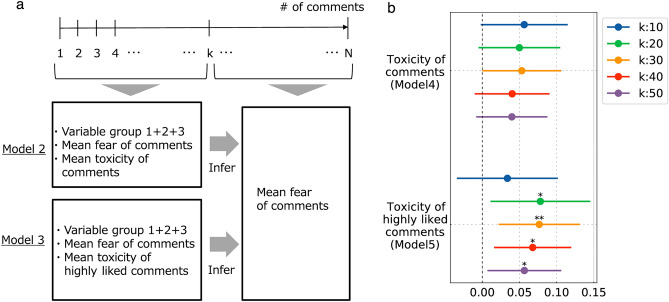
Measuring the association of the fear of early comments with the toxicity in later comments. (**a**) Illustration of the problem set. *N* comments in chronological order for a given video are divided into early and later halves, separated by *k*. Then, the mean fear in comments in the comment range is inferred by the variables noted in Model 6 and Model 7, respectively, and the coefficients are obtained. (**b**) Forest plots showing the coefficients of the fear in comments and the fear in highly liked comments, for $k=\{\mathrm{10,20,30,40,50}\}$. Only the coefficients for fear in highly liked comments are largely significant (3 out of 5 cases).

