# Supplementary material for: The impact of toxic trolling comments on anti-vaccine YouTube videos
Source: Sci Rep. 2024 Mar 1;14:5088. doi: 10.1038/s41598-024-54925-w (PMC10907339; doi:10.1038/s41598-024-54925-w)
Supplement: Supplementary file 1 — Supplementary Information. [file 41598_2024_54925_MOESM1_ESM.pdf]

Supplementary Material

The Impact of Toxic Trolling Comments on Anti-vaccine YouTube Videos

Kunihiro Miyazaki[1*], Takayuki Uchiba[2], Haewoon Kwak[1], Jisun An[1] and Kazutoshi Sasahara[3]

[1*]Luddy School of Informatics, Computing, and Engineering, Indiana University Bloomington, Bloomington, IN, USA.
[2]Sugakubunka Co., Ltd., Tokyo, Japan.
[3]School of Environment and Society, Tokyo Institute of Technology, Tokyo, Japan.

*Corresponding author(s). E-mail(s): kunihirom@acm.org;
Contributing authors: takayuki.uchiba@sugakubunka.com; haewoon@acm.org; jisun.an@acm.org; sasahara.k.aa@m.titech.ac.jp;

Topic	Count	Topic words
virus	130	virus cell disease body patient system blood infection brain problem
government	288	trump president state country government money company right case party
others	244	world life movie right game point one god part let
route	382	friend child family house home life room school girl week
child	313	child disease measles vaccination health case doctor autism parent study
school	185	school person life point student child part parent work anything

Table 1 Top six topics video transcripts. Count is the number of videos belonging to each topic. Topic words are the representative words of each topic.

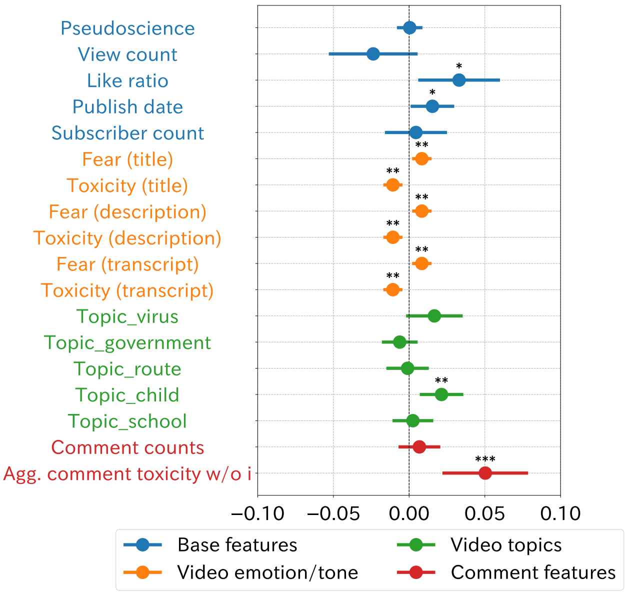

Fig. 1 **Robustness check for video-level regression.** We run a comment-level regression of fear for comment "i" with the same features as Fig. 2, as well as the average toxicity score for all other comments but "i." We here clustered standard errors at the video level to account for interdependencies across comments. The coefficient of variables with 95% CIs. The stars indicate the p values of the t-test: *** for $p < 0.001$, ** for $p < 0.01$, and * for $p < 0.05$. The model intercept parameter is not shown.

	Model1	Model2	Model3	Mean	Std
const	0.419***	0.198***	0.149*	-	-
View count	-0.073*	0.026	-0.068	0.6	0.15
Like ratio	-0.202***	-0.079	-0.052	0.91	0.13
Pseudoscience	0.033**	0.011	0.008	0.19	0.39
Publish date	0.029	0.046	0.017	0.8	0.18
Subscriber count	0.021	-0.022	-0.009	0.67	0.18
Fear (title)	-	0.141***	0.138***	0.11	0.21
description_fear	-	0.058**	0.065***	0.2	0.3
Fear (subtitle)	-	0.079***	0.082***	0.21	0.3
Toxicity (title)	-	0.037	0.011	0.07	0.15
description_toxicity	-	0.005	-0.008	0.06	0.13
Toxicity (subtitle)	-	-0.024	-0.068*	0.18	0.19
Topic_virus	-	0.084***	0.099***	0.08	0.27
Topic_government	-	-0.009	-0.011	0.18	0.38
Topic_route	-	0.017	0.011	0.27	0.44
Topic_child	-	0.096***	0.098***	0.19	0.4
Topic_school	-	0.031	0.037	0.12	0.32
Toxicity (comment)	-	-	0.177***	0.42	0.15
Comment counts	-	-	0.049	0.6	0.3
R-squ.	0.059	0.231	0.258	-	-
N	1,072	707	707	-	-

Table 2 Regression results of mean fear of comments at the video level. Model 1, 2, and 3 indicates the ablation study on independent values. Model 1 corresponds to the video's base features. In addition to them, Model 2 covers the video's emotion-related features. Model 3 corresponds to all the features including the comment features. Columns indicate the three models with different sets of variables and the mean and standard deviation (Std) for each variable. The rows indicate each variable, the coefficient of determination (R-square), and the number of samples (N). The cells are the coefficients in each model and the stars indicate the p values of the t-test: *** for $p < 0.001$, ** for $p < 0.01$, and * for $p < 0.05$.

Emotion	Coefficient
Fear	0.177***
Anger	0.575***
Disgust	0.542***
Joy	-0.511***
Neutral	-0.158***
Sadness	0.044
Surprise	-0.083**

Table 3 Coefficients of Toxicity (comment) and its significance in video-level regression analysis when the dependent variable emotion of fear is replaced to the other six emotions obtained in the RoBERTa model.
